# Obtaining long 16S rDNA sequences using multiple primers and its application on dioxin-containing samples

**DOI:** 10.1186/1471-2105-16-S18-S13

**Published:** 2015-12-09

**Authors:** Yi-Lin Chen, Chuan-Chun Lee, Ya-Lan Lin, Kai-Min Yin, Chung-Liang Ho, Tsunglin Liu

**Affiliations:** 1 Molecular Diagnostic Laboratory, Department of Pathology, National Cheng Kung University Hospital, Tainan, Taiwan; 2 Molecular Medicine Core Laboratory, Research Center of Clinical Medicine, National Cheng Kung University Hospital, Tainan, Taiwan; 3 Environmental Analysis Laboratory, Environmental Protection Administration, Executive Yuan, Taiwan; 4 Institute of Bioinformatics and Biosignal Transduction, National Cheng Kung University, Tainan, Taiwan

**Keywords:** metagenomics, 16S rDNA, next-generation sequencing (NGS), multiple primers, assembly, dioxin

## Abstract

**Background:**

Next-generation sequencing (NGS) technology has transformed metagenomics because the high-throughput data allow an in-depth exploration of a complex microbial community. However, accurate species identification with NGS data is challenging because NGS sequences are relatively short. Assembling 16S rDNA segments into longer sequences has been proposed for improving species identification. Current approaches, however, either suffer from amplification bias due to one single primer or insufficient 16S rDNA reads in whole genome sequencing data.

**Results:**

Multiple primers were used to amplify different 16S rDNA segments for 454 sequencing, followed by 454 read classification and assembly. This permitted targeted sequencing while reducing primer bias. For test samples containing four known bacteria, accurate and near full-length 16S rDNAs of three known bacteria were obtained. For real soil and sediment samples containing dioxins in various concentrations, 16S rDNA sequences were lengthened by 50% for about half of the non-rare microbes, and 16S rDNAs of several microbes reached more than 1000 bp. In addition, reduced primer bias using multiple primers was illustrated.

**Conclusions:**

A new experimental and computational pipeline for obtaining long 16S rDNA sequences was proposed. The capability of the pipeline was validated on test samples and illustrated on real samples. For dioxin-containing samples, the pipeline revealed several microbes suitable for future studies of dioxin chemistry.

## Background

Metagenomics has revolutionized microbiology by directly studying environmental microbes that are mostly unculturable [1,2]. Next-generation sequencing further advances this field because high-throughput data allow an in-depth examination of a complex microbial community [3,4]. Sequencing of phylogenetic marker genes (e.g. 16S rDNA) is a popular approach for identifying microbial species. However, it is challenging with NGS data because NGS sequences, often called reads, are relatively short [5]. For example, popular 454 and Illumina reads are ~400 and 250 bp, respectively, which are much shorter than the ~1500 bp 16S rDNA sequences. For short NGS reads, taxonomy classification is less confident [6]. Common classification tools (e.g. RDP classifier [7], SINA [8], and MG-RAST [9]) often determine taxonomy only to the genus or even higher levels for NGS reads.

To tackle the challenges of studying metagenomics using short NGS reads, it has been proposed that different 16S rDNA segments can be assembled into full-length 16S rDNA [10-12]. In two studies, 16S rDNA segments were extracted from whole genome shotgun sequencing data for assembly [10,11]. Although the approach is free of primer bias, only a tiny portion (~0.1%) of the data contained 16S rDNA segments. Another approach is to amplify whole 16S rDNA genes using a single primer followed by shotgun sequencing and assembly [12]. Like all targeted sequencing approaches, most of the data obtained can be used. However, primer bias can distort the microbial community structure significantly [13,14].

**Figure 1:**
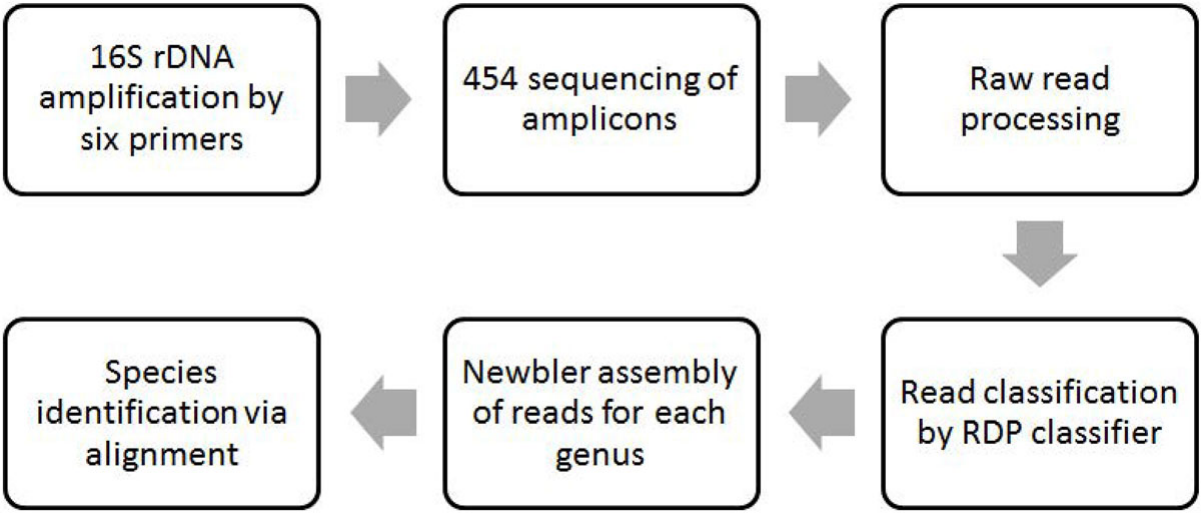
**Pipeline for obtaining long 16S rDNA for species identification**.

In this work, a pipeline was proposed to overcome the drawbacks of current approaches for obtaining long 16S rDNA sequences using NGS data. In the pipeline (Figure 1), multiple primers were used to amplify different 16S rDNA segments for sequencing. The NGS reads were then classified into genera, and reads of each genus were assembled into a long 16S rDNA sequence. This strategy maintained the advantage of targeted sequencing and reduced primer bias because it was less likely for a microbe to be missed by all primers. Another advantage was the ability to compare results using different primers for a more reliable conclusion. However, there were many practical challenges in implementation. For example, a 16S rDNA gene might not be amplified by all primers; therefore, a full-length 16S rDNA sequence might not be obtained. Moreover, short NGS reads could be misclassified, leading to false assembled sequences.

To assess the feasibility of the current pipeline, a proof-of-concept experiment was conducted on test samples containing some known bacteria. This permitted pipeline adjustments for generating accurate and long 16S rDNA sequences. After ensuring the capability of the pipeline, it was applied to real samples that were polluted with dioxins. To our knowledge, this is the first metagenomic study of dioxin-containing samples using NGS technology.

## Methods

### Sample preparation

Three test samples (S1-S3) and seven real samples (S4-S10) were prepared in this study (Table 1). S1 was a solution containing only four bacteria: *Legionella pneumophila, Chryseobacterium, Pseudomonas*, and *Bacillus. Legionella pneumophila *(BCRC 17854, from Food Industry Research and Development Institute, Taiwan) was purchased. The remaining bacteria in S1 were cultured from S2 and S3 using Tryptocase Soy Agar (TSA) plates. S2 was a regular soil sample, and S3 was sediment from a dioxin-polluted site in Taiwan [15]. Bacteria cultured from S2 were identified as *Chryseobacterium *and *Pseudomonas*, and from S3 as *Bacillus *using BioLog (BioLog GEN III microtest system, BioLog Inc., U.S.A.). Cultured bacteria were added back to the source samples, respectively, and all of them were added to S1. Note that the purchased *Legionella pneumophila *was also added to S2 and S3 as a control. Samples S4-S9 were obtained from various locations in the same dioxin-polluted site. S4 and S5 were from a pentachlorophenol (PCP) factory; S6 and S7 were sediments from a seawater pool; S8 and S9 were from another factory. These samples contained dioxins, heavy metals (mainly mercury), and chemicals in various concentrations [15]. S10 was collected from the exterior of the dioxin-polluted site. The order of dioxin concentrations in real samples was S4>S5>S6>S7>S8>S9>S10.

**Table 1:** Statistics of reads, genera, and contig lengths of ten samples.

Sample	Raw reads	Trimmed reads	Confident reads	Total genus	Confident genus	No. of contigs	**lcl/mrl**. ≥1.5	lcl/mrl ≥2	lc identity ≥97%
S1	31,035	29,170	27,423	8	4	4	4	3	4

S2	38,100	35,007	30,242	144	28	23	15	6	11

S3	39,881	35,456	10,909	191	62	53	28	12	27

S4	91,44	4,590	478	65	9	9	4	3	3

S5	25,314	16,857	3,059	218	38	34	24	12	14

S6	14,709	8,347	854	117	18	13	5	2	2

S7	13,810	8,262	983	128	20	17	4	3	4

S8	11,379	9,214	2,145	230	47	37	18	5	18

S9	9,924	8,277	1,374	193	33	30	13	5	16

S10	12,084	8,216	1,685	124	23	20	11	4	7

A confident read is one with a ≥80 classification score. A genus is counted when there is a confident read. A confident genus is one with ≥10 confident reads. Confident reads of each confident genus were assembled and the longest contig (lc) analyzed. The last column shows the number of longest contigs with a ≥97% alignment identity to the 16S rDNA references. Abbreviations: longest contig length (lcl), mean read length (mrl).

### Bacteria identification using BioLog

For S2 and S3, 10 g of samples were vigorously mixed with 100 mL phosphate buffered saline. The homogeneous mixture was allowed to stand for one minute. Supernatants (10 mL) were then taken for 10x serial dilution. For each dilution, 200 μL diluted solution were streaked onto a TSA plate and cultured for 48 hours at 30°C. Cultured bacteria were further grown on BioLog BUG+B agar for identification following the manufacturer's protocol. The BioLog system comprised 71 carbon sources and 23 chemical sensitivity tests for identifying microorganisms.

### DNA extraction and 16S rDNA amplification

From a minimum 1 g of soil, DNA was isolated using the PowerSoil^® ^DNA Isolation Kit (MO BIO Laboratories Inc., U.S.A.) according to manufacturer instructions. Purity and DNA yield were assessed using spectrophotometry (NanoDrop, Thermo Fisher Scientific, Canada) (Additional file 1: Table S1). Full-length 16S rDNAs were PCR-amplified using each of the six primer pairs reported previously [6]. Each 25 μL PCR reaction mixture consisted of genomic DNA (50 ng), 22 μL Super Therm Gold Master Mix (BIONOVAS Biotechnology Co., USA), 1 μL forward primer (10 uM), and 1 μL reverse primer (10 uM). PCR was performed in a G-Storm PCR machine (G-Storm, United Kingdom) with the following cycling conditions: an initial denaturation at 95°C for 6 min, 40 cycles of denaturation at 95°C for 30 s, annealing at 50°C for 1 min, and extension at 72°C for 1 min. A final extension was performed at 72°C for 10 min. PCR products were analyzed using 2% agarose gel electrophoresis. Sizes of the amplified 16S rDNA segments using six primers were estimated as 527 bp (8F-534R), 456 bp (343F-798R), 410 bp (517F-926R), 331 bp (784F-1114R), 491 bp (917F-1407R), and 443 bp (1099F-1541R), respectively.

### 454 sequencing

The 16S rDNA libraries were amplified via emulsion-PCR on a thermocycler (G-Storm, United Kingdom) according to the Roche 454 em-PCR amplification manual - Lib L (454 Life Sciences, U.S.A.). Products were sequenced in a GS Junior system (Roche Diagnostic, U.S.A.) at the National Cheng Kung University, Taiwan. Samples were barcoded and pooled (S1-S3, S4-S7, and S8-S10 in the first, second, and third runs, respectively) for sequencing. Because S4 and S5 did not have enough reads in the second run, the remaining S4 and S5 samples were added to the third run.

### Read classification and assembly

To avoid sequencing bias at primer binding sites, primer parts were removed from 454 raw reads by trimming the first and last 25 bp of all reads. Only trimmed reads as long as 200 bp were used for analysis. Reads were classified into genera using RDP Classifier [7] (v2.5, default parameters). A read with a ≥80 classification score was called a confident read. A genus containing at least 10 confident reads was called a confident genus. For each confident genus, all confident reads were assembled using Newbler [16] (v2.7, options: -ml 100 -mi 98). For a genus containing two or more distinct 16S rDNA genes, assembly might discontinue at the distinct regions, resulting in multiple contigs branching out from a base contig. In this case, the branching contig with a read coverage <10 (adjustable) was discarded. For each remaining branching contig, the base contig was copied and concatenated, leading to a longer contig. It was also possible that two slightly different 16S rDNA sequences were merged into one contig during assembly. When only one contig was output, constituting reads were examined for positions containing a minor nucleotide that appeared ≥10 times. A sequence pattern at recognized positions was kept if it also appeared ≥10 times. If more than one sequence pattern survived processing, the contig was duplicated, and each sequence pattern was introduced to the positions, resulting in multiple differing contigs.

### Species identification

For each confident genus, the longest assembled contig was aligned against microbial 16S rDNA sequences from the RDP database [7] (release 11) using BLAST (v2.2.27+, options: -evalue 0.01 -perc_identity 97). The best alignment, i.e., one with the highest score, was chosen and the corresponding species was considered a species of the genus if the alignment identity was ≥97%.

### Sanger sequencing

To validate identified bacteria, additional primers were designed (Additional file 1: Table S2) to amplify full-length 16S rDNA of *Legionella, Chryseobacterium*, and *Pseudomonas*. PCR cycling conditions were as follows: (1) at 95°C for 5 min; (2) 30 cycles at 95°C for 30 s, 50°C for 30 s, and 72°C for 30 s; and (3) 72°C for 10 min. Amplified products were purified using the FAVORGEN cleanup kit (Biotech Corp., Taiwan) and sequenced using the BigDye Terminator Sequencing Kit (Applied Biosystems, U.S.A.). Sequencing products underwent electrophoresis in an ABI PRISM 3500 genetic analyzer (Applied Biosystems). For each ambiguous base call, the flowgram was checked manually, and the base with a higher peak was chosen.

### Comparison of microbial communities

For each sample, reads were clustered into operational taxonomic units (OTUs) using the mothur package [17] (v1.32) as follows. First, reads were aligned (command: align.seqs, options: align=needleman, flip=t) to microbial 16S rDNA sequences from the RDP database. Reference sequences that contained more than 10 ambiguous nucleotides (N's) were filtered. Second, pairwise distances between reads were calculated (command: dist.seqs, option: cutoff = 0.1). Based on pairwise distances, reads were then clustered into OTUs (cluster option: method=furthest, cutoff = 0.03). Finally, the representative sequence of each OTU was determined (command: get.oturep).

Microbial communities were compared using Fast UniFrac [18] (v1.5.3). First, OTU representatives of all samples were collected and labeled. Second, representative sequences were aligned to 16S rDNA references, and their pairwise distances were calculated as in the OTU analysis. Third, a phylogenetic tree of these sequences was constructed using mothur (command: clearcut). Based on the tree, distances between communities were calculated using Fast UniFrac. Weighted measurement of distances, i.e., considering numbers of reads reflecting OTU representatives, was used. Distances between samples were visualized after principal component analysis; results were plotted using R package [19] (v3.0.1).

Microbial communities were also compared according to composition of microbes at six taxonomic levels: kingdom, phylum, class, order, family, and genus. For each level, percentages of confidently classified taxonomies were calculated for all real samples. The top 15 taxonomies with the highest mean percentages across all samples were shown in a stacked histogram. Note that Fast UniFrac analysis was conducted for each primer, whereas reads amplified using all primers were lumped together for calculating microbial compositions.

### *In-silico *evaluation

To evaluate *in-silico *sensitivity of six primers, known bacterial 16S rDNA sequences from the RDP database were used as amplification targets. For each primer, whether a 16S rDNA sequence could be amplified was determined using ePCR [20] (v2.3.9; fahash options: -w 3; re-PCR options: -n 2; insert size: 450-550 for primer A and E, 350-500 for B and F, 350-450 for C, and 250-350 for D). Some 16S rDNA sequences, especially the shorter ones, could not be amplified simply because they did not extend to the primer binding sites. To consider such limitation, the span of each 16S rDNA sequence was determined via its multiple sequence alignment. The sensitivity of each primer was then defined as ratio of the amplifiable sequences to sequences extending to the primer binding sites.

For each 16S rDNA sequence, the amplified segments were further assembled if they overlap by at least 10 bp; the number of assembled sequences as long as 1000 bp was counted. In addition, the number of 16S rDNA sequences that covered the binding sites of four consecutive primers (e.g., A-D or B-E) was counted. For those 894701 sequences, assembly of the amplified segments might reach 1000 bp or longer. The fraction of long assembled sequences was then calculated as ratio of the two numbers.

For comparing primer bias, full-length 16S rDNA sequences were first selected. A 16S rDNA reference was considered as full-length if it covered the position of the primer (27F 5'-AGAGTTTGATCCTGGCTCAG-3'; 1492R 5'-GGTTACCTTGTTACGACTT-3') used in a previous study [12]. The sensitivity of that primer on the 156890 full-length 16S rDNA sequences was determined again using ePCR. In addition, the fraction of full-length 16S rDNA sequences that could be amplified by at least one of the six primers was calculated based on the ePCR results.

## Results

### Capability of the pipeline on test samples

#### a. Bacteria broth (S1)

Among test samples, S1 was the least complex and contained only four bacteria: *Legionella pneumophila, Chryseobacterium, Pseudomonas*, and *Bacillus*. Only *Legionella *was known to the species level because it was purchased. The remaining bacteria were cultured from S2 and S3, and their identities were experimentally determined to the genus level.

RDP classifier put the 29170 trimmed reads into 58 genera (data not shown), including the four known bacteria. Only eight genera remained when 1747 (6.0%) non-confident reads (with a <80 classification score) were excluded (Table 1). Among the eight genera, known bacteria had a much higher number (>2000) of confident reads than other false bacteria (≤6). Thus, a confident genus was defined as one with at least 10 confident reads; only confident genera were further analyzed.

For each known bacterium, all six primers (A-F) successfully amplified the 16S rDNA gene (Additional file 1: Table S3). However, amplifications were not uniform across primers. For example, 38.3% of the confident reads of *Legionella *were amplified using primer C, whereas only 3.1% were amplified using primer B. For each confident genus, confident reads were assembled using Newbler. Except for *Bacillus*, Newbler generated a contig at least twice as long as the mean read length (Table 1). Contigs of *Chryseobacterium *and *Pseudomonas *reached 1483 and 1468 bp (Table 2), respectively, close to the full length of a common 16S rDNA gene.

**Table 2:** Statistics of contigs, Sanger sequences, and their alignments.

**(a) Contig lengths of four known bacteria in three test samples**.				
**Sample**	**Length of longest contig (bp)**			

	***Legionella***	***Chryseobacterium***	***Pseudomonas***	***Bacillus***
	
S1	780	1483	1468	479

S2	1143	1052	1193	N.A.

S3	1487	N.A.	N.A.	845

**(b) Results of BLAST alignments between each contig and the Sanger sequence.**				

**Genus**	**Sanger (bp)**	**Identity (%); mismatch; gap**		
		
		**S1**	**S2**	**S3**

*Legionella*	832	99.8; 0; 1	99.6; 0; 2	99.6; 0; 2

*Chryseobacterium*	1126	100; 0; 0	100; 0; 0	N.A.

*Psuedomonas*	1529	100; 0; 0	99.8; 1; 1	N.A.

To validate the assembly, 16S rDNA of the four bacteria, excluding *Bacillus*, were subjected to Sanger sequencing. The 16S rDNA sequences obtained were 832, 1126, and 1529 bp for *Legionella, Chryseobacterium*, and *Pseudomonas *(Table 2), respectively. Contigs of *Chryseobacterium *and *Pseudomonas *were 100% identical to corresponding Sanger sequences (Table 2). The *Legionella *contig differed from the Sanger sequence by only one gap, which may be a homopolymer error of 454 sequencing.

To identify the species of the four bacteria, contigs were aligned to 16S rDNA references from the RDP database using BLAST. The species corresponding to the best hit with a ≥97% alignment identity was assigned to each bacterium. Of these hits, the *Legionella *contig was best aligned to the 16S rDNA of *Legionella pneumophila*, which was indeed the purchased species. The other three bacteria were identified as *Chryseobacterium sp. WG4, Pseudomonas monteilii*, and *Bacillus licheniformis*.

#### b. Soil with spiked-in bacteria (S2)

Sample S2 was soil with three spiked-in bacteria: *Legionella pneumophila, Chryseobacterium*, and *Pseudomonas*. RDP classifier put the 30242 confident reads into 144 genera, of which 28 genera were confident (Table 1). Among the 29976 confident reads in the 28 confident genera, 28482 (95.0%) were classified to the genera of the three spiked-in bacteria. For the three bacteria, Newbler generated contigs longer than 1000 bp (Table 2). The *Chryseobacterium *contig was 100% identical to the Sanger sequence. For *Pseudomonas*, only one mismatch and one gap occurred in the 1176 bases aligned to the Sanger sequence. For *Legionella*, only two gaps were observed in the 808 aligned bases. The three bacterial species identified using BLAST were the same as in S1.

Only 1492 confident reads were classified to the remaining 25 genera, and Newbler generated contigs twice as long as the mean read lengths for only three genera (Table 1): *Clostridium sensu stricto, Sporacetigenium*, and *Bacillus*. The three genera ranked 3^rd^, 4^th^, and 6^th ^in number of confident reads (containing 198, 186, and 88 reads), respectively. Although lengths of the 16S rDNA sequences were not doubled in most cases, they were increased by at least 50% for 12 of the 25 genera (Table 1). Species identification was still possible for some assembled contigs. Among the 25 genera, the species of eight genera could be determined because their contigs were aligned with a ≥97% identity.

#### c. Sediment with spiked-in bacteria (S3)

Sample S3 was sediment with two spiked-in bacteria: *Legionella pneumophila *and *Bacillus*. Among the 35456 trimmed reads, only 10909 (30.8%) reads were classified confidently (Table 1). Compared to S2, a lower fraction (26.1% v.s. 95.0%) of confident reads in confident genera were from spiked-in bacteria. Assembled contigs of spiked-in *Legionella *and *Bacillus *were 1487 and 845 bp, respectively (Table 2). The *Legionella *contig was fully aligned to the Sanger sequence with only two gaps (Table 2).

Among the remaining 60 genera, Newbler generated a contig at least 1.5 and 2 times longer than the mean read length for 28 and 12 genera (Table 1), respectively. Sorted by number of confident reads, all top 15 genera (with ≥143 reads) had a contig at least 50% longer than the mean read length. For some genera, fewer confident reads still resulted in a long contig. For example, the 22 confident reads of *Clostridium XI *were assembled into a 795 bp contig, two times longer than the mean read length (356 bp). Of the 60 microbes, the species of 26 could be determined using assembled 16S rDNA sequences.

### Performance of the pipeline on dioxin-containing samples

The seven real samples were sequenced in two runs. Total numbers of raw reads, 57920 and 38700, were lower than the 109232 raw reads of the first run on test samples. Base qualities of the two runs were also lower (Additional file 2: Figure S1). Compared to test samples, percentages of confident reads (ranging from 10.2% to 23.3%, Table 1) were lower. For all seven real samples, 188 confident genera were found, and Newbler generated contigs for 160 (85%) genera. Among the 160 contigs, about half (79) were 50% longer and 34 were two times longer than the mean read length (Table 1); eight were longer than 1000 bp. RDP classifier assigned all contigs to the same genera of the constituting reads. More importantly, classification scores of the assembled contigs were higher than mean confidence scores of reads for 145 (90.1%) of the 160 genera. Among the 160 genera, microbial species could be determined for 64 genera (Table 1).

### Primer bias

For a real sample, the bias of a primer was estimated as the percentage of confident genera that would be missed if only data of the primer were used. A genus was considered missed by a primer if no confident read of the primer was from the genus. For real samples, primer C was the least biased and missed only about 5% of the genera on average (Table 3). In contrast, primers B and E were the most biased and missed about 40-60% of the genera.

**Table 3:** Primer bias, i.e., percentage of confident genera that would be missed by each of the six primers.

Primer	S1	S2	S3	S4	S5	S6	S7	S8	S9	S10
A	0%	36%	42%	**11%**	45%	39%	50%	32%	33%	30%

B	0%	54%	47%	44%	58%	50%	80%	55%	58%	**26%**

C	0%	7%	15%	**11%**	**5%**	**0%**	**10%**	**2%**	**9%**	**13%**

D	0%	39%	29%	**33%**	**18%**	44%	30%	**21%**	21%	30%

E	0%	75%	35%	44%	42%	56%	40%	47%	36%	52%

F	0%	21%	35%	56%	21%	**17%**	**15%**	**21%**	**12%**	**26%**

No. of genus	4	28	62	9	38	18	20	47	33	23

For real samples, the two least biased primers are shown in bold.

### Comparison of the microbial communities in real samples

**Figure 2:**
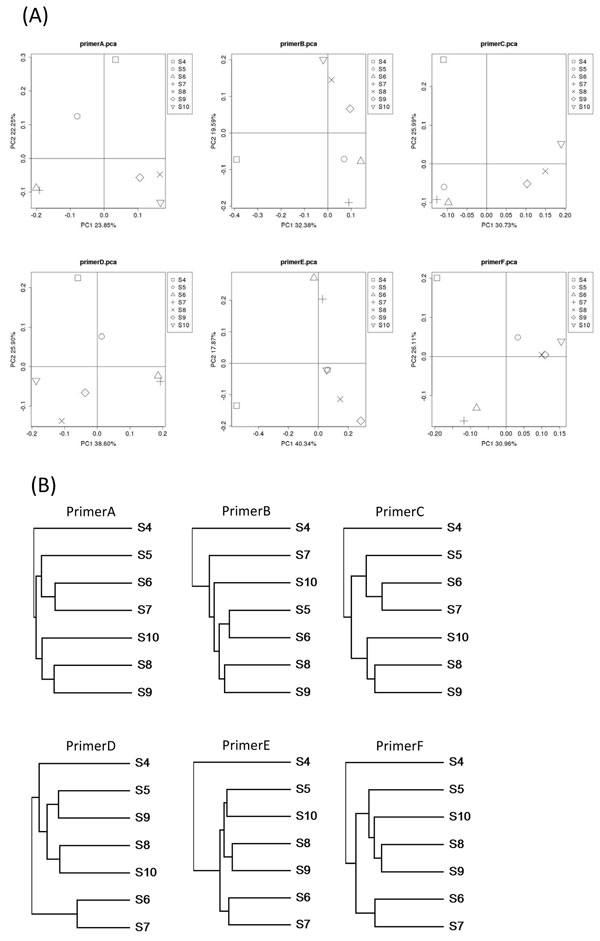
**Distances between real samples and their clustering**. (a) Principal components of distances between seven microbial communities in real samples by UniFrac. (b) Clustering of the seven communities using full distances.

To illustrate an additional benefit of using multiple primers, microbial communities in all real samples were compared using Fast UniFrac for each primer. For five of the six primers, S6 and S7 (two sediment samples) clustered together (Figure 2). Thus, it was more reliable to conclude similarity between the two sediment samples. Similarly, S8 and S9, which were from the same factory, were grouped together for five of the six primers. Interestingly, S4 and S5 were from the same factory but did not cluster together for all six primers (Figure 2). In fact, S4 was rather distinct from all other samples. Coincidently, the dioxin concentration in S4 was the highest among real samples. This motivated further examination of the microbial community in S4.

### Microbial species in real samples

In terms of microbial compositions, S5 was the closest to S4 at the order, family, and genus level (Additional file 2: Figure S2). To compare communities at the species level, the assembled contig of each confident genus of S4 was aligned to the corresponding contig of other real samples. Consistently, eight of the nine genera in S4 were also present in S5 (Table 4), whereas at most three were present in other samples. Between S4 and S5, five species were likely the same because alignment identities were ≥97.0%. Species of the remaining three genera in S4 should have been different from those in S5 as the alignment identities were <97%.

**Table 4:** Alignment identity of nine contigs in S4 to corresponding contigs in samples S5-S10.

Genus in S4	S5	S6	S7	S8	S9	S10	Candidate species
*Hydrogenophaga*	99.8%	-	-	98.1%	-	-	*Uncultured Beta Proteobacteria*

*Gracilimonas*	99.5%	-	-	-	-	-	*Uncultured Bacteroidetes*

*Ignavibacterium*	90.6%	-	-	92.1%	93.1%	-	N.A.*

*Sulfuriculvum*	100.0%	-	-	-	-	-	*Sulfuriculvum kujiense DSM 16994*

*Hylemonella*	96.2%	-	-	-	-	-	*Uncultured bacterium*

*Acinetobacter*	100.0%	-	-	100.0%	-	99.9%	*Acinetobacter bereziniae*

*Gp21*	93.3%	-	-	-	-	-	*Uncultured Acidobacteria*

*Phycisphaera*	98.89%	99.41%	-	-	-	-	*Uncultured bacterium*

*OD1_genera_incertae_sedis*	-	-	-	-	-	-	*Uncultured bacterium*

The last column shows candidate species of the seven genera in S4. *Alignment identity <97%.

### *In-silico *evaluation of the current pipeline using MiSeq reads

Currently, Illumina MiSeq can provide nearly 600 bp sequences (see Discussion), which are longer than amplicons of the six primers. Therefore, most amplicons can be sequenced entirely using MiSeq and the performance of the current pipeline relies mainly on sensitivity of the six primers. Using known bacterial 16S rDNA sequences as amplification targets, *in-silico *sensitivities of the six primers ranged from 77.3% (primer F) to 95.6% (primer C) (Table 5). Most (99.3%) of the 16S rDNA sequences could be amplified by at least one of the fix primers. Moreover, 633240 assembled sequences were as long as 1000 bp, accounting for 70.8% of the 16S rDNA references.

**Table 5:** *In-silico *sensitivity of the six primers.

Primer	No. of 16S rDNA sequences covering the primer position	No. of amplifiable sequences	Percentage
A	468049	411649	87.9%

B	2140848	1873932	87.5%

C	1877993	1796209	95.6%

D	1787993	1424757	79.7%

E	1003626	915216	91.2%

F	61634	47650	77.3%

At least one	2472276	2455930	99.3%

## Discussion

### Requirements for obtaining long 16S rDNA

Our results suggest several requirements for obtaining long 16S rDNA sequences using the current pipeline. The first essential condition is that 16S rDNA of a microbe can be amplified by several "neighboring" primers. Two primers are neighbors when their amplicons overlap by at least 40 bp, thus enabling assembly in the current pipeline. For the six primers A-F, only consecutive primer pairs, for example, A and B or D and E, are neighbors [6]. Primer design is clearly important in the pipeline. A good primer set is expected to (1) capture 16S rDNA genes of most microbes (i.e., be highly universal), (2) cover whole 16S rDNA genes, and (3) give amplicons shorter than sequencing reads. The primer set used in the current pipeline meets the last two conditions, and the universality of some primers has been confirmed in a previous study [6]. Moreover, all six primers successfully amplified 16S rDNA for all samples in this study. Note that designing another primer set may be necessary for a different sample, especially when some primers cannot amplify 16S rDNA, which has been observed (data not shown). It is possible to use more primers to increase the number of neighboring primers. However, greater loading of experiments and comparative analyses are required. Moreover, performance must be further evaluated.

The second requirement is enough data for presenting most amplified 16S rDNA segments. The sufficiency of data is governed by three factors: uniformity of primer amplification, species abundance, and sequencing depth. Amplifications using multiple primers are usually not uniform, which was indeed observed for the known bacteria in this study. On average, about 200 reads were needed to double the length of 16S rDNA for a genus in real samples of this study. In other words, our pipeline only doubled the lengths of 16S rDNA for 34 of the 188 confident genera in real samples because there were not enough reads for the majority of genera. For these genera, the scarcity of reads suggested rarity of the species. It is clear that greater read depth is required for revealing less abundant species. The current pipeline applies 454 sequencing, the throughput of which is only moderate among NGS platforms. Illumina MiSeq is a promising alternative for the present pipeline because its data throughput is greater than 454. Although MiSeq reads are shorter than 454 reads (300 v.s. ~400 bp), it is possible to merge the so-called paired-end reads that overlap into longer single reads. Our *in-silico *evaluation showed that using MiSeq reads, the current pipeline could provide 16S rDNA sequences as long as 1000 bp for 70.8% of the known reference sequences.

Third, 16S rDNA reads must be classified to the correct genera with confidence. This requirement is essential for controlling false positives, which was illustrated by the 54 false genera found in S1 if a classification score of ≥80 was not required. Setting a minimal number of confident reads further controlled false positives. For example, requiring 10 confident reads eliminated all false genera in S1. For a greater amount of data, it is more appropriate to require a minimal fraction of reads. These requirements also imply that the current pipeline is more suitable for known microbes than for novel microbes because novel 16S rDNAs are often classified with a lower confidence. This could partially explain lower percentages of confident reads in real samples than those in test samples as more novel microbes were expected to exist in real samples.

Finally, the current pipeline can distinguish two or more non-rare species in the same genus. When 16S rDNA sequences of two species in the same genus are distinct (e.g., with a <98% identity), assembly usually stops at the boundary of the distinct regions, resulting in several short contigs. Fortunately, Newbler keeps track of connections between contigs (Additional file 2: Figure S3a), and the information was used for recovering two long 16S rDNA sequences in the current pipeline. When two 16S rDNA sequences from the same genus differ by only few bases, Newbler merges the two sequences into one contig. The current pipeline searched the detailed assembly of each contig for positions showing more than one non-rare base (Additional file 2: Figure S3b). Once found, the contig was duplicated, and distinct sequence patterns were assigned to those positions. With these processes, 16 of the 160 genera in real samples were found to contain two or more different 16S rDNA sequences.

### Comparison with other approaches

The approach that extracts 16S rDNA reads from whole genome shotgun sequencing data [10,11] is less promising for real samples of this study using 454 sequencing. If whole genome shotgun sequencing were to be conducted perfectly on a real sample, the number of 16S rDNA reads in the data would be about 100 (0.1%*100,000). Consequently, the number of reads confidently classified to a genus would be less than 10 because reads of most genera constituted less than 10% of the total reads (Additional file 2: Figure S3). If sample pooling and non-perfect sequencing were considered, the read number would be even smaller or drop to zero for most genera. It is noteworthy that the whole genome approach is more promising if Illumina sequencing is applied. However, the performance of our pipeline is also expected to increase using Illumina data. Thus, a comprehensive comparison is still needed.

For comparison with the targeted approach using one single primer [12], primer bias was estimated as follows. For each of the six primers and the primer used in the previous targeted approach, the percentage of 16S rDNA references that could be amplified was determined using ePCR. Only 0.2% of the full-length 16S rDNA sequences were missed by all six primers (data not shown). In contrast, 7.4% were missed by the primer in the previous targeted approach. Despite greater primer bias, the previous targeted approach is advantageous once a 16S rDNA gene can be amplified. Because amplified genes are under shotgun sequencing, there will be no concern for non-uniform amplification across multiple primers. As a result, fewer reads are needed for obtaining a long 16S rDNA sequence.

### Environmental impacts on the pipeline

On test samples, the current pipeline successfully generated accurate and near full-length 16S rDNAs for three of four known bacteria. The assembled contigs of each known bacteria (except Bacillus) in different test samples aligned with each other with 100% identity (data not shown). This indicates consistency of the pipeline across these environments. That is, the presence of other bacteria or low-concentration chemicals did not affect the assembly of major species.

In contrast, the environments of dioxin-containing samples did impact the pipeline because the base quality and number of reads were lower compared to the test run. This could be another reason for the lower percentage of confident reads in real samples. The lower base quality for dioxin-containing samples was not accidental as quality returned to the original level in the next run of sequencing another bacterial broth (Additional file 2: Figure S1). It is possible that contamination started to interfere with the microbial community when the concentration was above a cutoff, which is supported by a report of a dose-dependent effect of PCP on a microbial community [21].

Lower sequencing quality could affect assembly. For example, 28 of the 188 confident genera in real samples did not have an assembled contig because the sequence identity in overlapping regions fell below 98%. Despite the lower sequencing quality, the current pipeline still lengthened 16S rDNA by at least 50% for half of the microbes, and some 16S rDNAs reached 1000 bp.

### Putative microbes related to dioxin

At the kingdom level, the percentage of archaea in S4 was the highest among all real samples (Additional file 2: Figure S2). Because dioxin concentration was also highest in S4, abundant archaea in S4 may be linked to dioxin or related chemicals. A putative related chemical is PCP because S4 was from a PCP factory and dioxins are by-products of PCP manufacturing. It has been shown that methanogenic archaea are selected during PCP degradation in reactors [22-24]. Although methanomicrobia were not abundant in confident reads of S4, it became one of the major archaea classes if non-confident reads were included (data not shown). There are two possible explanations for non-confident classifications: (1) methanogenic archaea were novel, and (2) sequencing quality was low. The second possibility was excluded because the mean quality of S4 reads was not the lowest among real samples (Additional file 2: Figure S4). Thus, novel methanogenic archaea likely existed in S4. In fact, the fraction of putative novel microbes was the highest in S4 at the kingdom, phylum, and class level (Additional file 2: Figure S2).

Along this line, the minimal confidence requirement for classification was dropped and all archaea reads were re-classified via alignments to NCBI 16S rDNA database [25] using BLAST. Alignments with an identity (number of matched bases divided by read length) at least 0.9 were selected and corresponding taxonomies were assigned to the reads; other reads were considered as unclassified. Two genera, *Candidatus_Nitrosoarchaeum *and *Nitrosopumilus*, were relatively more abundant in S4 compared to other samples (Additional file 1: Table S4). Interestingly, *Nitrosopumilus maritimus *has been implicated in the dioxin degradation pathway in KEGG [26]. The enzymes 4-oxalocrotonate tautomerase and pyruvate carboxyltransferase produced by *N. maritimus *facilitate dioxin degradation. These results suggest that archaea species also play a role in dioxin degradation and deserve further exploration, which can be relatively novel and complementary because most current studies of biodegradation of dioxin focus on bacteria.

At the order level, Burkholderiales were most abundant in the two samples from the PCP factory; its concentrations in other samples were relatively low. Some *Burkholderia *species have been shown to be resistant to PCP [21] and involved in dioxin degradation [27]. However, the corresponding family in S4 and S5 was Comamonadaceae instead of Burkholderiaceae, the family of the *Burkholderia *species. This might be explained by different primers used in our and the previous studies, with the result that different Burkholderiales species were captured. Interestingly, a Comamonadaceae genus, *Comamonas*, has been reported to degrade PCP [28] and dibenzofurans [29]. However, the reported genus was different from our major genus *Hydrogenophaga*. Nevertheless, a *Hydrogenophaga *species has been shown to degrade polychlorinated biphenyls, which are dioxin-like compounds [30].

## Conclusions

The current pipeline could generate accurate and long 16S rDNA sequences when there were sufficient 454 reads from those genera. For dioxin-containing samples, the pipeline lengthened 16S rDNA by at least 50% for about half of the non-rare species and generated 16S rDNAs longer than 1000 bp for some species. Our data also revealed several microbes (e.g., *Nitrosopumilus *and *Hydrogenophaga*) that may be involved in the chemistry of dioxin or PCP.

## List of abbreviations

NGS: next-generation sequencing; PCP: pentachlorophenol;

## Competing interests

None declared.

## Authors' contributions

KMY collected real samples. YLC extracted DNAs, amplified 16S rDNA segments, and performed 454 sequencing with the assistance of YLL. CCL analyzed data and helped prepare the manuscript. CHL and TL designed and directed the study. TL finalized the work and wrote the manuscript. All authors read and approved the final manuscript.

## Supplementary Material

Additional file 1 **Table S1**. DNA concentrations and quality controls of ten samples. **Table S2**. Additional primers for amplifying full-length 16S rDNA of three known bacteria. **Table S3**. Percentages of confident reads amplified by six primers (A-F) for four known bacteria in sample S1. **Table S4**. Percentages of archaea genera (relative to all archaea reads) in real samples. Click here for file

Additional file 2 **Figure S1**. Mean quality at each base position of 454 reads obtained in four runs of sequencing. Only the first three runs are for this study and the fourth run is for a bacterial broth without contamination. **Figure S2**. Compositions of microbes in the seven real samples. At each level, the 15 most abundant classifications (by mean percentage across all samples) are shown from bottom to top and the rest are denoted by "others". The space between a stack top and 100% represents non-confident reads. **Figure S3**. Post-processing of assembly for identifying more than one non-rare species in a genus. (a) Distinct segments of two different 16S rDNA sequences, corresponding to contig2 and contig3, result in a bubble structure of contig connection. Because both contigs are supported by ≥10 reads, the assembly is rearranged into two 16S rDNAs: contig1-contig2-contig4 and contig1-contig3-contig4. (b) Two different sequence patterns in an assembled contig are observed. If both patterns appear more than 10 times, the contig is duplicated and the two patterns are assigned to the positions. **Figure S4**. Mean quality at each base position of 454 reads of seven real samples. Click here for file

## Declaration

Publication charges for this article have been funded by Ministry of Science and Technology, Taiwan (MOST 103-2221-E-006-180).

This article has been published as part of *BMC Bioinformatics *Volume 16 Supplement 18, 2015: Joint 26th Genome Informatics Workshop and 14th International Conference on Bioinformatics: Bioinformatics. The full contents of the supplement are available online at http://www.biomedcentral.com/bmcbioinformatics/supplements/16/S18.
