# Supplementary material for: Obtaining long 16S rDNA sequences using multiple primers and its application on dioxin-containing samples
Source: BMC Bioinformatics. 2015 Dec 9;16(Suppl 18):S13. doi: 10.1186/1471-2105-16-S18-S13 (PMC4682383; doi:10.1186/1471-2105-16-S18-S13)
Supplement: Additional file 1 — Table S1. DNA concentrations and quality controls of ten samples. Table S2. Additional primers for amplifying full-length 16S rDNA of three known bacteria. Table S3. Percentages of confident reads amplified by six primers (A-F) for four known bacteria in sample S1. Table S4. Percentages of archaea genera (relative to all archaea reads) in real samples. [file 1471-2105-16-S18-S13-S1.pdf]

Additonal file 1

Supplementary Tables

Sample	DNA concentration	260/280nm
S1	64.5 ng/µl	1.29/0.671 (1.92)
S2	67.5 ng/µl	1.358/0.75 (1.81)
S3	36.2 ng/µl	0.724/0.201 (3.6)
S4	52.2ng/µl	1.044/0.2 (5.2)
S5	11.2ng/µl	0.224/0.056 (4)
S6	75.9ng/µl	1.518/0.474 (3.2)
S7	91.1ng/µl	1.822/0.56 (3.25)
S8	33.3ng/µl	0.666/0.214 (3.1)
S9	67ng/µl	1.34/0.536 (2.5)
S10	67ng/µl	1.34/0.609 (2.2)

Table S1. DNA concentrations and quality controls of ten samples.

Legionella pneumophila	
70F	GTCTAGCTTGCTAGACAGATGGCGA
1251R	CGGCGAAGGGGCGACCTGGAGCAAATC
Chryseobacterium	
66F	AGATCTTTCGGGATCTT
1235R	TACACAGCGATGTGATGCAAATCTCGAAAG
Pseudomonas	
47F	AAGTCGAGCGGATGACGGGAGCTTGCTCCTT
1251R	GAGGTGGAGCTAATCTCACAAAACCG

Table S2. Additional primers for amplifying full-length 16S rDNA of three known bacteria.

Genus	A	B	C	D	E	F
Legionella	10.5%	3.1%	38.3%	21.4%	7.0%	19.7%
Pseudomonas	19.5%	5.1%	18.0%	17.5%	5.7%	34.2%
Chryseobacterium	18.9%	6.5%	34.0%	33.7%	6.7%	0.1%

Bacillus	5.0%	6.5%	34.2%	9.2%	4.1%	41.0%

Table S3. Percentages of confident reads amplified by six primers (A-F) for four known bacteria in sample S1.

Genus\sample	S4	S5	S6	S7	S8	S9	S10
Candidatus_Nitrosoarchaeum	10.32	1.46	0	0.17	1.32	0	0
Nitrosopumilus	10.32	1.46	0	0.17	1.32	0	0
Nitrososphaera	0	0	0	0	1.75	0	1.28
Methanosarcina	0	0	0	0	0.44	0	0
Methanolobus	0	0	0	0	0.44	0	0
Halolamina	0	0.24	0	0	0	0	0
Halogranum	0.13	0	0	0	0	0	0
Haloferax	0.13	0	0	0	0	0	0
Halopelagius	0.13	0	0	0	0	0	0
Natronomonas	0.13	0	0	0	0	0	0
unclassified	78.84	96.84	100	99.67	94.74	100	98.72

Table S4. Percentages of archaea genera (relative to all archaea reads) in real samples.
