# Supplementary material for: Obtaining long 16S rDNA sequences using multiple primers and its application on dioxin-containing samples
Source: BMC Bioinformatics. 2015 Dec 9;16(Suppl 18):S13. doi: 10.1186/1471-2105-16-S18-S13 (PMC4682383; doi:10.1186/1471-2105-16-S18-S13)
Supplement: Additional file 2 — Figure S1. Mean quality at each base position of 454 reads obtained in four runs of sequencing. Only the first three runs are for this study and the fourth run is for a bacterial broth without contamination. Figure S2. Compositions of microbes in the seven real samples. At each level, the 15 most abundant classifications (by mean percentage across all samples) are shown from bottom to top and the rest are denoted by "others". The space between a stack top and 100% represents non-confident reads. Figure S3. Post-processing of assembly for identifying more than one non-rare species in a genus. (a) Distinct segments of two different 16S rDNA sequences, corresponding to contig2 and contig3, result in a bubble structure of contig connection. Because both contigs are supported by ≥10 reads, the assembly is rearranged into two 16S rDNAs: contig1-contig2-contig4 and contig1-contig3-contig4. (b) Two different sequence patterns in an assembled contig are observed. If both patterns appear more than 10 times, the contig is duplicated and the two patterns are assigned to the positions. Figure S4. Mean quality at each base position of 454 reads of seven real samples. [file 1471-2105-16-S18-S13-S2.pdf]

Additional file 2

Supplementary Figures

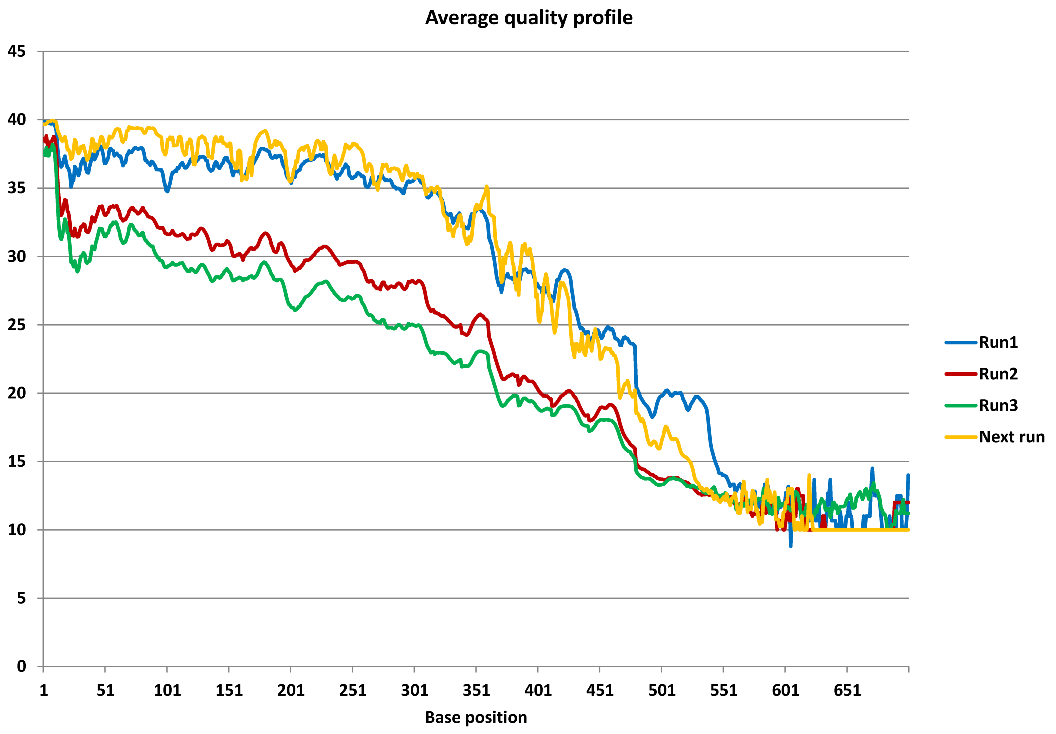

Figure S1. Mean quality at each base position of 454 reads obtained in four runs of sequencing. Only the first three runs are for this study and the fourth run is for a bacterial broth without contamination.

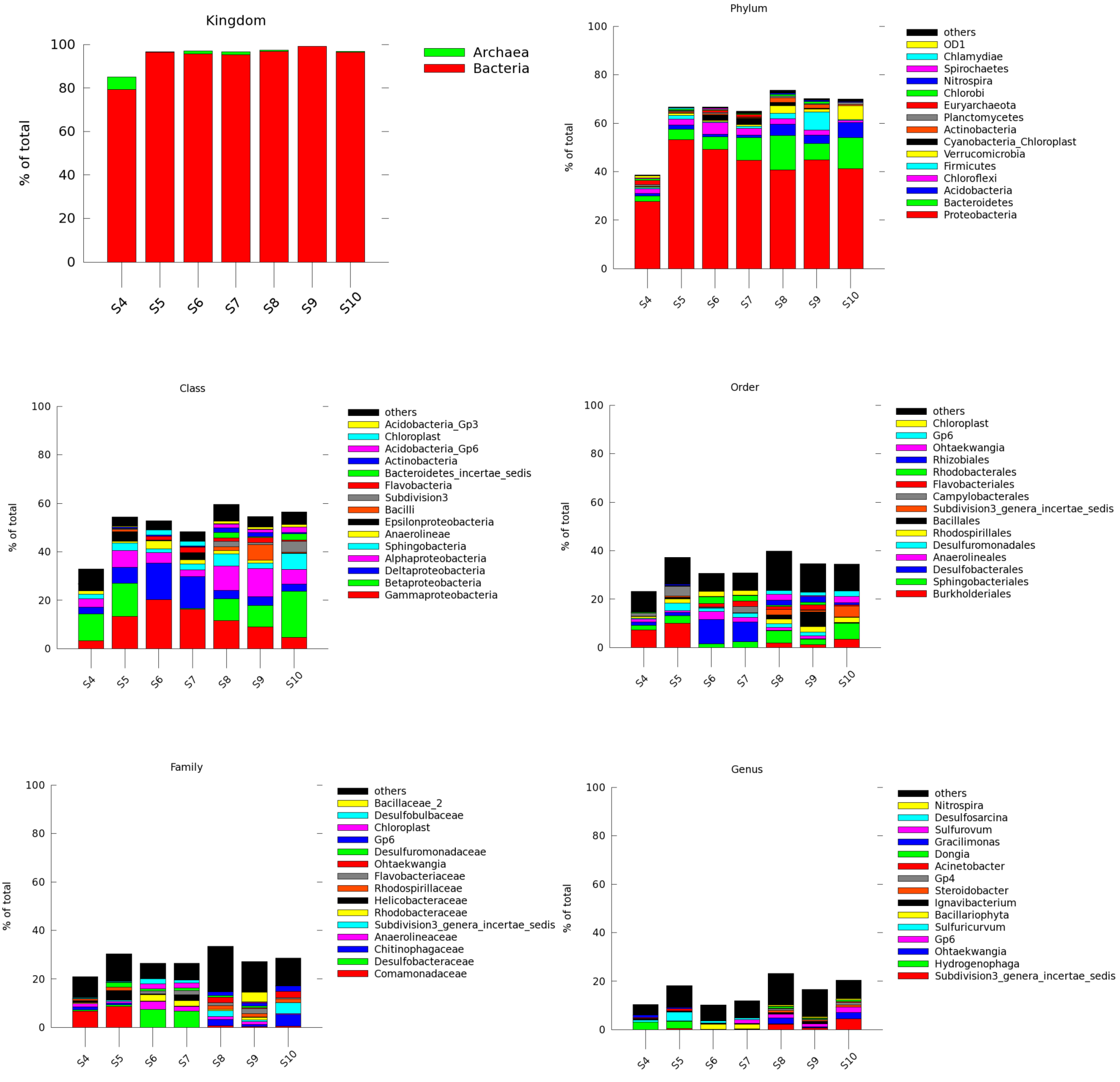

Figure S2. Compositions of microbes in the seven real samples. At each level, the 15 most abundant classifications (by mean percentage across all samples) are shown from bottom to top and the rest are denoted by “others”. The space between a stack top and 100% represents non-confident reads.

(a)

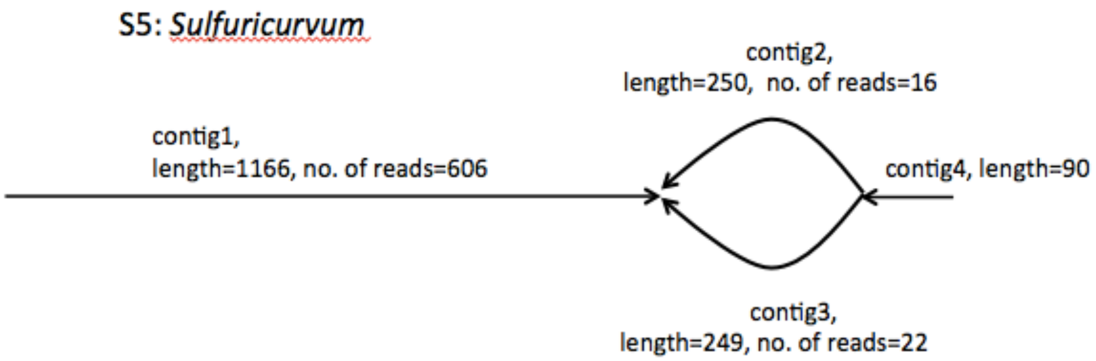

(b)

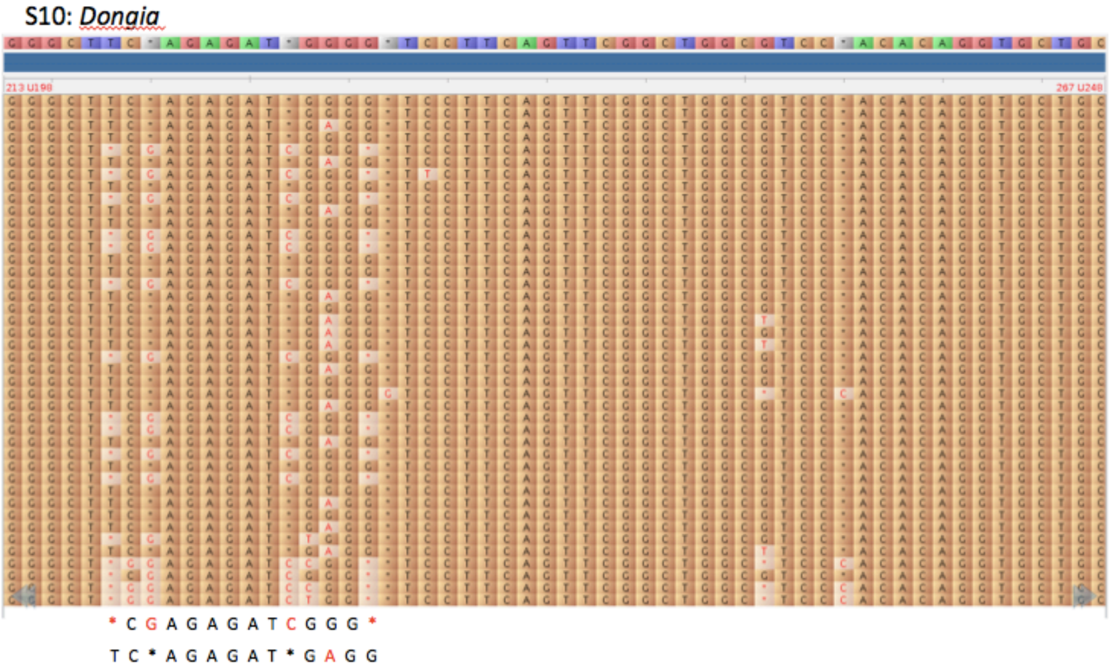

Figure S3. Post-processing of assembly for identifying more than one non-rare species in a genus. (a) Distinct segments of two different 16S rDNA sequences, corresponding to contig2 and contig3, result in a bubble structure of contig connection. Because both contigs are supported by ≥10 reads, the assembly is rearranged into two 16S rDNAs: contig1-contig2-contig4 and contig1-contig3-contig4. (b) Two different sequence patterns in an assembled contig are observed. If both patterns appear more than 10 times, the contig is duplicated and the two patterns are assigned to the positions.

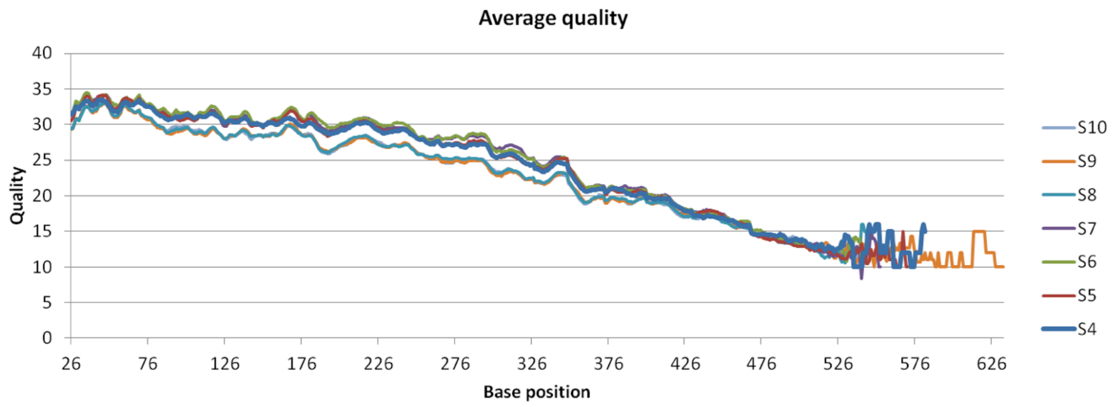

Figure S4. Mean quality at each base position of 454 reads of seven real samples.
